# Chilblain-like lesions onset during SARS-CoV-2 infection in a COVID-19-vaccinated adolescent: case report and review of literature

**DOI:** 10.1186/s13052-022-01296-5

**Published:** 2022-06-13

**Authors:** Roberto Paparella, Luigi Tarani, Enrico Properzi, Francesco Costantino, Chiara Saburri, Roberta Lucibello, Antonio Richetta, Alberto Spalice, Lucia Leonardi

**Affiliations:** 1 Department of Maternal and Child Health and Urology, Sapienza University of Rome, Rome, Italy; 2 Unit of Dermatology, Department of Internal Medicine and Medical Specialties, Sapienza University of Rome, Rome, Italy

**Keywords:** COVID-19, Chilblain-like lesions, Case report, SARS-CoV-2 vaccination, Children

## Abstract

**Background:**

COVID toes or chilblain-like skin lesions have been widely reported during COVID-19 pandemic. Most cases were described in patients with negative microbiological tests for SARS-CoV-2, therefore the possible relationship with SARS-CoV-2 infection, as well as with the nowadays broadly available mRNA-based vaccination, has not been fully elucidated.

**Case presentation:**

We here describe the case of a 14-year-old male who developed chilblain-like skin eruptions during SARS-CoV-2 infection despite two mRNA-based vaccine doses and review the clinical and epidemiological characteristics of chilblain-like lesions as a cutaneous presentation of COVID-19 in children.

**Conclusions:**

Most children and adolescent with COVID toes have a mild or asymptomatic SARS-CoV-2 infection. Our report aims to highlight the possible onset of these skin lesions in vaccinated children, if infection has occurred, and the potential use of systemic corticosteroids as a first line treatment. Additional evidence is required to better understand SARS-CoV-2 infection and cutaneous manifestations in children and determine the relationship between chilblain-like lesions and COVID-19 vaccination.

## Background

Coronavirus disease 2019 (COVID-19) pandemic is caused by the novel severe acute respiratory syndrome coronavirus (SARS-CoV-2), first isolated in Wuhan, China, in December 2019 and subsequently rapidly spreading worldwide [1]. In addition to systemic symptoms, several cutaneous manifestations related to COVID-19 infection have been observed, including perniosis-like or chilblain-like lesions (CLLs), also called COVID toes [2], characterized by vascular lesions due to microthrombosis and endothelial inflammation [3].

Despite an increased incidence of CLLs during COVID-19 pandemic, the true relationship between SARS-CoV-2 infection and this type of skin manifestation is still uncertain, since the majority of cases occurred with no evidence of positive reverse transcriptase-polymerase chain reaction (RT-PCR) test or serum antibodies [4]. However, given that the second wave of COVID-19 pandemic has been followed by a second outbreak of CLLs, a link not only of an epidemiological but also a causal nature is very likely [5]. Moreover, an increasing number of reports of CLLs shortly after SARS-CoV-2 vaccination is observed [6]. Here we present the case of a 14-year-old asymptomatic COVID-19 male showing COVID toes despite two vaccine doses, with a short review of what is currently known about CLLs as a cutaneous presentation in the context of pediatric COVID-19.

## Case presentation

**Fig. 1:**
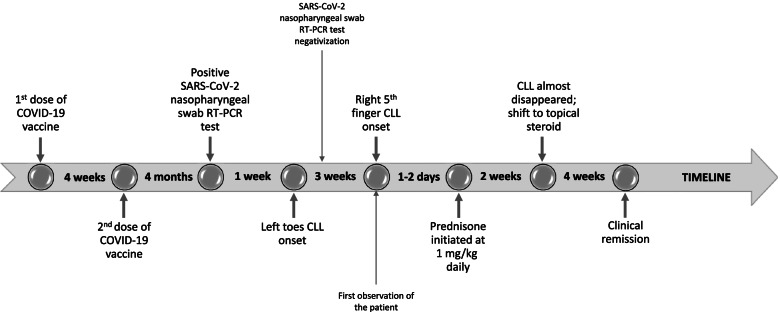
Timeline portraying the patient’s clinical history

A 14-year-old male patient presented to our hospital with CLLs on his left toes. His medical history was unremarkable, except for two epileptic seizures at the age of 13 with non-specific electroencephalography abnormalities and brain magnetic resonance imaging showing a retrocerebellar arachnoid cyst. There was no family history of cutaneous or autoimmune conditions. No recent exposure to cold temperatures, traumatic injuries or drug intake were reported. He had completed primary COVID-19 vaccination cycle (two doses of Comirnaty mRNA-based vaccine) four months before CLLs onset Fig. 1.

**Fig. 2:**
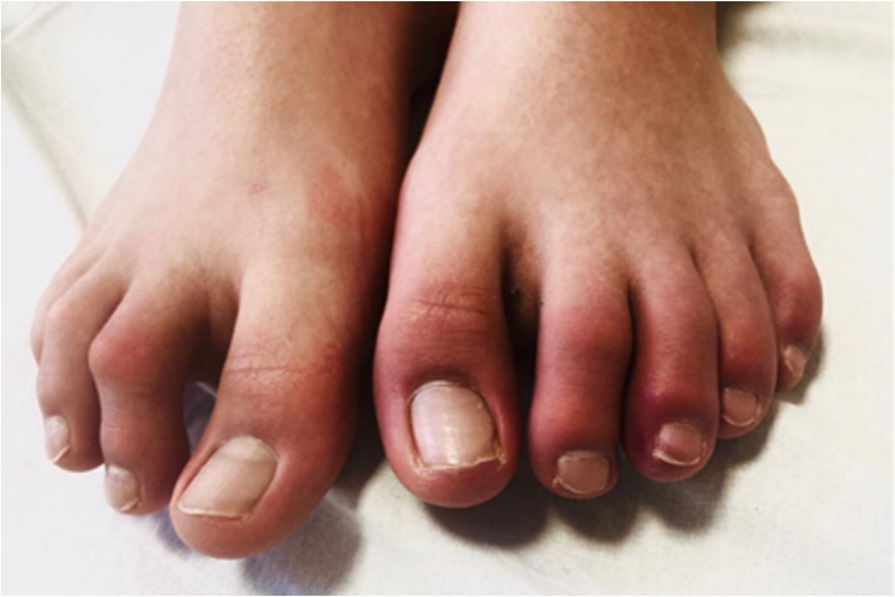
Characteristic aspect of chilblain‐like lesions of left toes

SARS-CoV-2 nasopharyngeal swab RT-PCR test resulted positive approximately one month before our first evaluation, being performed because of a household close contact. During the first days of his asymptomatic SARS-CoV-2 infection he developed swollen, erythematous lesions of left toes Fig. 2 associated with nighttime localized itching and walking difficulties.

The patient was admitted to our pediatric outpatient service after the onset of a new, similar, painful lesion on the right fifth finger, three weeks after the onset of the initial, persisting cutaneous manifestations. At this time, SARS-CoV-2 nasopharyngeal swab tested negative. Physical examination was otherwise normal. Complete blood count, coagulation tests, liver and renal and pancreatic function tests, blood electrolytes, and urinalysis were all within normal limits. Erythrocyte sedimentation rate, C-reactive protein, and serum C3 and C4 levels were normal. The cutaneous findings were compatible with COVID-19 CLLs, with diagnostic validation by a dermatologist. Oral prednisone was initiated at 1 mg/kg daily, leading to a quick improvement of the symptoms and attenuation of the skin lesions. Lupus anticoagulant, anti-β2 glycoprotein I IgG and IgM and anti-cardiolipin IgG and IgM, antinuclear antibody and extractable nuclear antigen panel were negative. The capillaroscopic examination revealed “non-specific changes”.

**Fig. 3:**
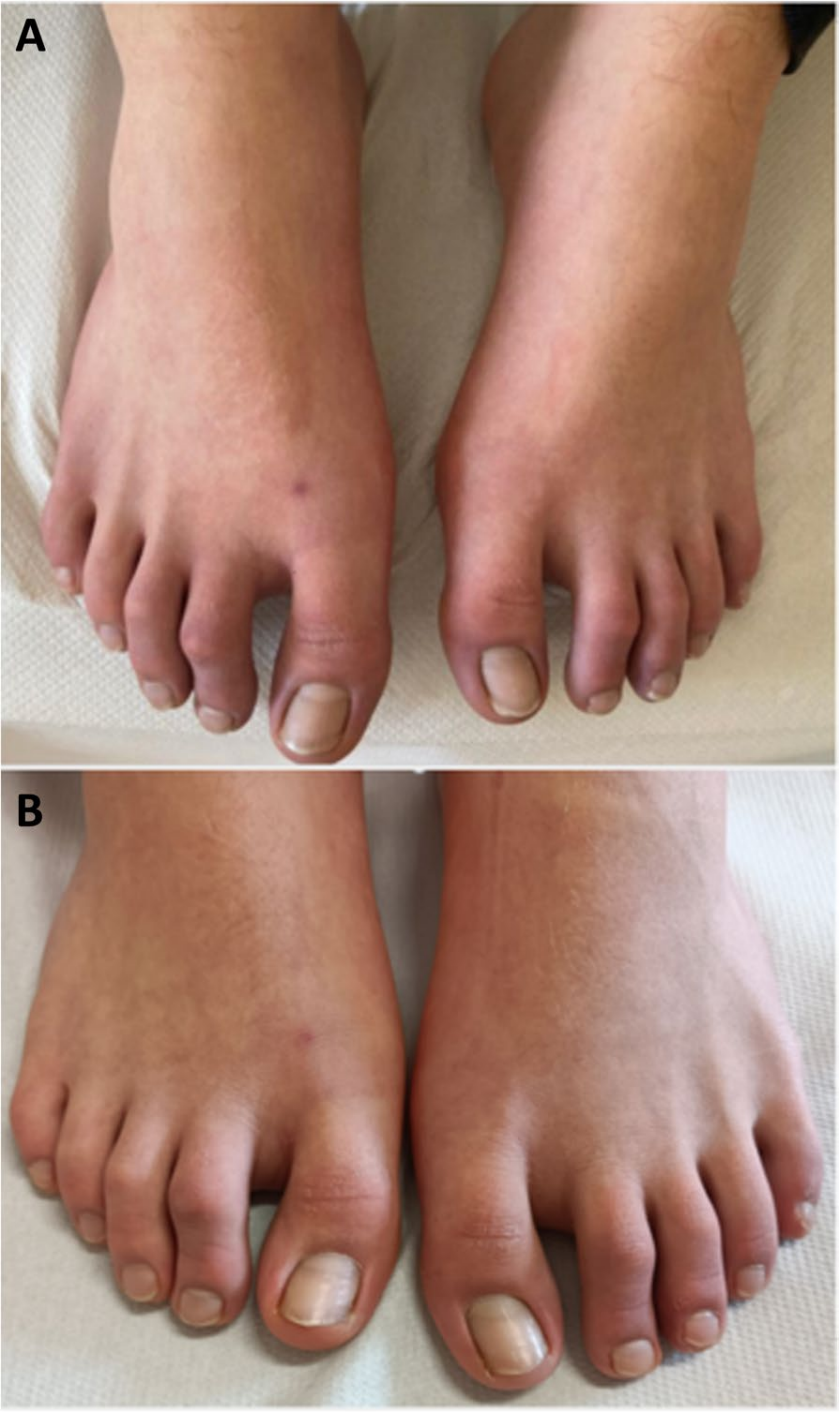
Almost complete resolutions of skin lesions after two (**A)** and six weeks (**B**)

Two weeks later, the patient showed a good clinical response; skin lesions had almost disappeared Fig. 3A and no symptoms were reported, allowing gradual systemic steroid withdrawal and shift to topical treatment. At the last follow-up evaluation, six weeks after our first observation, clinical remission was confirmed Fig. 3B.

## Discussion and conclusions

During the COVID-19 pandemic, numerous pediatric patients reporting CLLs with suspected SARS-CoV-2 infection were described. It was postulated that negativity of testing could be probably explained by the low viral load and infectivity at the time of the skin lesions, since CLLs are generally a late manifestation of a mild or asymptomatic disease [7]. Moreover, during the first COVID-19 wave, most of suspected SARS-CoV-2-associated CLLs were unconfirmed because of unavailability of RT-PCR testing. CLLs affect late childhood age, usually with a favorable prognosis and an indolent course, with a duration of weeks to months. Despite negativity of RT-PCR and serology testing, the presence of concomitant typical COVID-19-related symptoms or a positive contact history, in particular household viral exposure [8], makes the association between skin lesions and SARS-CoV-2 infection very likely. Our patient, indeed, had confirmed COVID-19 and CLLs, as already described in literature [9].

CLLs affect dorsal aspect of the toes, lateral sides of the feet, soles and less often fingers; clinical manifestations are variable, as they could present as dusky erythematous and edematous macules or plaques, purpuric lesions, or blisters formation, associated to pain, itching or no symptoms [10]. They share pathologic features with idiopathic and autoimmune-related chilblains: vacuolar interface dermatitis with necrotic keratinocytes, dermal edema, perivascular and perieccrine sweat gland lymphocytic inflammation, and frequent vascular changes (endothelialitis, microthrombi, fibrin deposition, and vascular immune deposits) [10–12].

The exact pathogenic mechanisms of CLLs in COVID-19 are still matter of debate. They resemble idiopathic chilblains, but are usually unrelated to classical risk factors such as exposure to cold or humid weather [13]. Some authors suggest that CLLs might also represent an indirect consequence of the COVID-19 pandemic due to lifestyle changes derived from community containment and lockdown measures [14]. The decreased physical activity and being barefoot during quarantine may have played a role in the pathogenesis of these lesions [15], although this hypothesis apparently poorly befits the third COVID-19 wave.

Type-I interferon (IFN-1) signaling is also involved in CLLs pathogenesis [7]. In pediatric patients, microangiopathic modifications with skin eruption and vasculitic neuropathic pain features are secondary to the strong IFN-1 response, which causes downregulation of other cytokines, not leading to a cytokine storm [16]. These findings contribute to explain why COVID-19-induced CLLs occur during mild or asymptomatic SARS-CoV-2 infections, in contrast to the different pathogenesis of thrombotic-related acral-ischemia with hypercoagulopathy state and elevated D-dimer levels in severely ill COVID-19 patients [17].

Another intriguing issue is the relationship between CLLs and SARS‐CoV‐2 vaccination. Our report represents a peculiar case of CLLs in a vaccinated patient with ongoing SARS-CoV-2 infection. Literature reports suggest that these lesions are not idiopathic, but rather represent an immunologic response to SARS-CoV-2, showing a positive IFN-1 signature [18]. Whilst during first pandemic wave children were not yet vaccinated, due to the recent surprising observations of CLLs following COVID-19 mRNA-based vaccination, it is tempting to speculate that vaccination might not be protective towards these late cutaneous manifestations if infection has occurred. In addition, a few cases of CLLs onset in vaccinated patients with not confirmed COVID‐19 infection have been reported [6].

Guidelines for the specific treatment of COVID-19 induced chilblains are still lacking. The management of idiopathic chilblains focuses primarily on the avoidance of unprotected exposure to cold conditions, not applicable to CLLs due to the different pathogenetic mechanisms. Other mainstay pharmacologic therapies include corticosteroids and calcium channel blockers [19, 20]. Oral cinnarizine showed as well good results in patients with COVID-19-associated CLLs, probably due to its antihistaminic and calcium channel blocking properties [21]. A successful combination of nitroglycerin with enoxaparin has also been reported [22].

In conclusion, CLLs are a peculiar dermatological finding in COVID-19 disease. Most pediatric patients with COVID toes have a mild or asymptomatic SARS-CoV-2 infection, implying that these cutaneous manifestations are the prerogative of children with a favorable disease course. Of note our case confirms that CLLs also occur in vaccinated children and that systemic corticosteroids might be a first line treatment for this COVID-19 sequela. Since there is currently limited information on the pathogenesis and the treatment of CLLs, further studies are required to update the body of knowledge about the mechanisms of SARS-CoV-2 infection in children, with regard in particular to dermatological manifestations, and the relationship between CLLs and SARS‐CoV‐2 vaccination.

## Data Availability

Data sharing not applicable to this article as no datasets were generated or analyzed during the current study.

## Abbreviations

CLLs: Chilblain-like lesions
COVID-19: Coronavirus disease 2019
IFN-1: Type-I interferon
RT-PCR: Reverse transcriptase-polymerase chain reaction
SARS-CoV-2: Severe acute respiratory syndrome coronavirus
